# Genome tagging project: tag every protein in mice through ‘artificial spermatids’

**DOI:** 10.1093/nsr/nwy136

**Published:** 2018-11-13

**Authors:** Jing Jiang, Meng Yan, Dangsheng Li, Jinsong Li

**Affiliations:** CAS Center for Excellence in Molecular Cell Science, Shanghai Institute of Biochemistry and Cell Biology, Chinese Academy of Sciences, China

The Human Genome Project (HGP), launched in 1990 and finished in 2004, has not only provided the complete human genome sequence of more than 2.85 billion nucleotides and evidence of 20 000–25 000 protein-coding genes [1], but has also been making huge impacts on biomedical research. One major task of the post-genomic era is to develop the definitive catalog of protein-coding genes, and illustrate proteins’ *in vivo* dynamic localization and physical interaction.

In the past decade, with the rapid development of bioinformatics methodologies, increasingly accurate catalog of protein-coding genes is emerging, with the most recent release containing a total of 21 232 [2]. However, the description of protein properties *in vivo* is still a challenge, especially at a genome-wide scale, mainly due to the difficulties in systematically producing reliable antibodies for the specific recognition of individual proteins. A generic solution to this problem is to genetically label the protein of interest with a tag, including fluorescent tags for *in vivo* visualization and affinity tags for the identification of interactions. Due to the advantages of efficient homologous recombination in yeast, protein tagging has been very successful and nearly all protein-coding genes have been tagged at their endogenous genomic loci, enabling global analysis of protein expression, localization and complexes using standard tag-based assays in yeast [3–5]. Genome-scale tagging has also been employed in *Caenorhabditis elegans* to generate a platform containing tagged worms covering about 73% of the proteome using a system called bacterial artificial chromosome (BAC) TransgeneOmics, in which the tag-coding sequence is inserted into a fosmid as transgenes that include all the important coding and regulatory sequences of a gene [6]. Similarly, a fly genome-wide fosmid library of 10 000 green fluorescent protein-tagged clones was generated recently and used to produce a total of 880 transgenic lines for protein localization analysis [7]. In mammals, BAC TransgeneOmics has also been successfully used in human tissue culture cells and mouse embryonic stem cells (ESCs) [8]. Nevertheless, it is extremely difficult to use the high-throughput approach to label protein-coding genes in mammals at the organismal level, such as in the mouse, which is the favored experimental mammal for biomedical studies, restricting large-scale protein analysis just to the cellular level. Moreover, the BAC transgene-based tag strategy may not recapitulate the physiological expression of some proteins due to the random insertion of the transgenes. One potential strategy to endogenously tag every protein in mice is to introduce a tag-coding sequence into the protein-coding gene in ESCs through conventional gene-targeting procedures, followed by the generation of tagged mice via the injection of tagged ESCs into blastocysts for chimera construction and germline transmission. However, chimera formation and germline transmission are always the rate-determining steps of this strategy, thus greatly impeding its large-scale application. Therefore, construction of a genome-wide tag-knock-in mouse library is still an unmet need and seems a formidable challenge for the biological research community.

**Figure 1.**
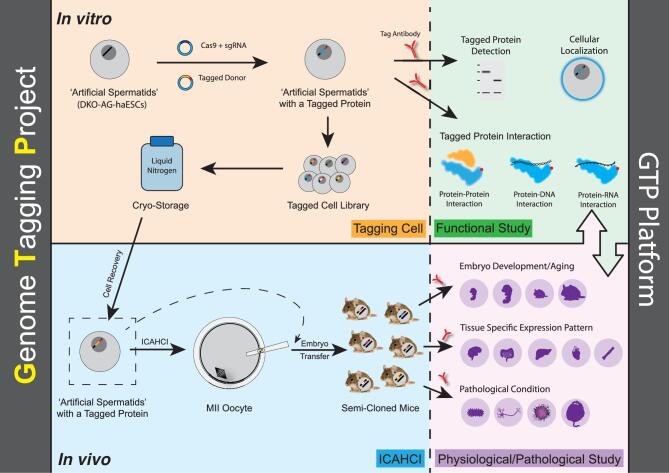
The workflow of the ‘artificial spermatid’-mediated Genome Tagging Project. Colored bars represent different protein-coding genes. Red bar represents the protein tag. ICAHCI, intracytoplasmic AG-haESC injection.

Recently, the emergence of two state-of-the-art technologies, clustered regularly interspaced short palindromic repeats (CRISPR)-Cas9-based genome editing and the generation of gene-modified semi-cloned (SC) mice from androgenetic haploid ESCs (AG-haESCs), has made the genome-wide tagging of protein-coding genes in mice an achievable scientific objective. CRISPR-Cas9 technology, which originated from the bacterial adaptive immunity system, can rapidly edit a genome with high efficiency and specificity through Cas9-mediated DNA cleavage at specific sites guided by single-guide RNAs (sgRNAs), resulting in DNA modifications by endogenous DNA repair systems [9]. With exogenously supplied DNA, CRISPR-Cas9 can induce precise gene editing at the targeted site, thus enabling efficient in-frame insertions of a tag-coding sequence. AG-haESCs are derived from haploid blastocysts with only the paternal genome [10]; after genetic removal of H19-DMR (differentially DNA methylated region) and IG-DMR, these cells, designated as DKO-AG-haESCs or ‘artificial spermatids’, can efficiently support the generation of SC mice after injection into oocytes (intracytoplasmic AG-haESC injection, ICAHCI) [11]. Importantly, the genetic manipulation of ‘artificial spermatids’ *in vitro* can produce SC pups with expected genetic traits at high efficiency. We thus reason that the ‘artificial spermatids’ could be used to precisely tag protein-coding genes at a genome-wide scale *in vitro* through the use of CRISPR-Cas9 technology, and that the resulting library of ‘artificial spermatids’, each with a specific protein tag, could be further used as a sperm replacement to produce a library of tagged mice in one step upon injection into oocytes. The combined application of ‘artificial spermatids’ and CRISPR-Cas9 has several advantages over the existing technologies for tagging genome-wide protein-coding genes in mice. First, ‘artificial spermatids’ enable *in vitro* genetic manipulation and genotyping analysis, leading to SC pups with a uniform tag and avoiding the mosaicism caused by direct zygote injection of CRISPR-Cas9. Second, ‘artificial spermatids’ are feasible for the design and selection of the best tag and insertion site for a specific protein-coding gene in culture, leading to SC pups with a suitable tag for endogenous protein analysis. Third, a library of ‘artificial spermatids’ with a tagged protein could be maintained *in vitro* and be compatible with long-term cryo-storage, and tagged mice could be generated at any time according to scientific needs (Fig. 1). In short, we can handle everything in liquid culture of ‘artificial spermatids’ before they are used to produce mice in one step, thus saving time, cost and space, and ensuring that tagging every protein in mice could be completed in a reasonable time frame.

The idea of tagging all proteins in mice using ‘artificial spermatids’, later designated as the Genome Tagging Project (GTP), was first conceived at a brainstorming meeting on molecular cell science held in May 2017 by the Shanghai Institute of Biochemistry and Cell Biology (SIBCB), an internationally renowned institution famous for the successful *in vitro* synthesis of insulin in the 1960s. In the year since the meeting, the SIBCB has been making great efforts to promote the GTP by organizing more meetings to discuss the scientific significance and organization of the project, granting the GTP team the first ‘bucket of gold’ to tag 50 proteins in ‘artificial spermatids’ and later establishing a center (the GTP Center) to execute the project at the end of 2017. Meanwhile, the Shanghai Municipal Commission for Science and Technology has supported the GTP by funding a pilot project to tag 500 cancer-related proteins in ‘artificial spermatids’ and produce 100 tagged mice by the end of June 2019. We believe that through this pilot project, the standard of operation for producing tagged products will be established for the genome-wide tagging efforts that will follow. Meanwhile, the GTP resource platform will be established for sharing resources with scientists all over the world, which will stimulate both national and international collaborations.

In the first version of the GTP (GTP 1.0) (Fig. 1), including the pilot project, we propose to choose the HA tag for protein tagging, due to its short peptide sequence, which does not appear to interfere with the bioactivity or biodistribution of the recombinant protein, and its diverse applications in standard protein assays. The goal of GTP 1.0 is to tag every protein in ‘artificial spermatids’ with the HA tag and produce corresponding mice covering most of genes with human homologs. By April 2019, a total of 474 protein-coding genes have been tagged with the HA tag in ‘artificial spermatids’, leading to stable cell lines in which proteins could be detected using an anti-HA antibody in 238 cell lines via western blotting analyses. Importantly, through ICAHCI technology, we have obtained 134 tagged mouse lines, which could be further used to describe the protein expression, localization and interactome during development using HA antibody-based assays. While we are confident in fulfilling the goal within a reasonable time period, some technical issues should be noted. First, the insertion site of the tag for each protein (N-terminal, C-terminal or internal sites) should be carefully analyzed. We propose that, in general, C-terminal tagging would be the first choice given that it is less likely to interfere with protein localizations. Second, the existence of different protein isoforms encoded by the same gene should be taken into consideration. Our first choice is to tag the longest isoform of each protein in GTP 1.0. Third, certain drawbacks of the CRISPR/Cas9 technology need to be attended to, such as off-target effects and a lack of suitable sgRNAs for certain target sites.

With the GTP platform (Fig. 1), we can employ standard HA tag antibody-based assays to investigate a group of proteins at the same time, which will enable the precise description of protein expression and localization patterns, and protein–protein, protein–DNA and protein–RNA interactions in development/aging, physiological and pathological conditions. In the meantime, the GTP platform promises to elevate protein studies from single-protein-based pipelines to a strategy based on grouped proteins, which may lead to unexpected discoveries that cannot be revealed by regular methods. Moreover, we believe that efforts to systematically tag the mouse proteome will promote the development of new tools and approaches that will accelerate biomedical research.
